# Investigating the Impact of Washing Cycles on Silver-Plated Textile Electrodes: A Complete Study

**DOI:** 10.3390/s20061739

**Published:** 2020-03-20

**Authors:** Valentin Gaubert, Hayriye Gidik, Nicolas Bodart, Vladan Koncar

**Affiliations:** 1 GEnie et Matériaux TEXtiles (GEMTEX) Laboratory, F-59100 Roubaix, France; hayriye.gidik@yncrea.fr (H.G.); vladan.koncar@ensait.fr (V.K.); 2 École Nationale Supérieure des Arts et Industries Textiles (ENSAIT), F-59100 Roubaix, France; 3 UCLille, Hautes Etudes Ingénieur (HEI)—YNCREA, F-59000 Lille, France; 4 Engineering, University of Lille, F-59650 Villeneuve d’Ascq, France; 5 Petit Bateau, 115, Rue du Lieutenant Pierre Murard, F-10000 Troyes, France; nbodart@fr.petitbateau.com

**Keywords:** smart textile washability, silver-plated nylon electrodes, textrodes, e-textile

## Abstract

Although market prediction for smart textiles in the coming years is high, their washability will be among the main criteria for their mass adoption. Hence, the need to understand precisely how the washing process can damage them. Therefore, the best care instructions can be determined and serve as guidelines for smart textile manufacturers to control the quality of their smart garments as well as their customers to wash them cautiously. In this study, only the sensing part, silver-plated-nylon electrode sensors, is taken into account. To determine the chemical and the mechanical impacts of the machine-washing process separately and simultaneously, textile electrodes were put in different washing conditions: with and without bleaching agents, with and without mechanical constraints, etc. Then spectrophotometry, Scanning Electron Microscopy (SEM) and Thermogravimetric Analysis (TGA) were used to characterize these electrodes. Results show that liquid detergents should be preferred to powder ones. Indeed, the latter contain bleaching agents that tend to oxidize the silver layer, making it more vulnerable to the mechanical rubbings that tear off the silver layer progressively washes after washes. As a consequence, the silver-plated-nylon loses rapidly its conductivity so that the electrode is no longer able to sense biopotentials.

## 1. Introduction

Even though a great market success has been announced for many years, no smart garment has been a great mass-market success yet. In fact, even if a lot of e-textiles prototypes have been developed in laboratories since 2000 [1], only a few smart garments have already hit the market [2]. One of the main reasons is the fact that wearable textronic prototypes suffer from poor washability and reliability [3]. The need to study the impact of the washing process on textronics was underlined back in 2004 [4] and it is all the more relevant that smart textiles are on the edge of being commercialized [5]. However, few research papers have tackled this particular issue until now, although it can be noted that the washability aspect of recent developments is more clearly taken into account [6,7,8]. However, as noted by Zaman et al. [9], there is a lack of standardizations on the washing process to be followed, the detergent to use and the number of washing cycles required to claim that a smart textile is really washable. As a result, among the few studies that can be found in the literature on the subject, smart garment prototypes are claimed washable according to various standards: ISO 6330:2000 [10,11], ISO 105-C01 [12] or ISO15797 [11], method 61-2010 of the American Association of Textile Chemists and Colorists (AATCC, Table 1) [6]. Hence, the utmost importance of the current work of research groups to provide a consensus standard with normalized tests for assessing the launderability and reliability of e-textiles, which is complex due to their heterogeneous nature. Indeed, e-textiles generally consists of three main parts that merge textile and electronic with different levels of integration:

(1) Sensors that are able to record external stimuli such as the electrical activity of the heart at the surface skin (e.g., ECG). It is the most textile part and usually made with conductive yarns or highly flexible substrates obtained from different technologies (metal-plating, coating with conductive polymers, etc.).
(2) An electronic module that records and processes the signal. It is usually the cumbersome part of e-textile as electronic components such as batteries that are rigid and cannot be miniaturized to keep their minimal functionalities. Furthermore, it should be kept away from water, which can damage instantaneously and permanently all the electronics. That is why, from now on, this module is generally pluggable in order to be removed before the washing process. However, attempts to encapsulating them are promising [3].
(3) Circuitry/connectors, which are the bridge between the sensors and the electronic module. Conductive paths can be knitted seamlessly as well as sensing electrodes when using silver-plated yarn. They can also be embroidered with metallic yarns or printed with conductive inks. The snap pressures are generally made with stainless steel that withstand well the washing process.

This study focuses only on the sensor part and more specifically on the washability of silver-plated-nylon electrodes. Although there is a global consensus on the impact of the washing process, which is the loss of conductivity of the conductive materials, no matter their nature (conductive yarns [13], inks [14] or polymers like PEDOT [7,8,15]). In most of the studies, only the mechanical stress of the washing process was taken into account and made responsible for this loss of conductivity. However, mechanical stress is not the only potentially damaging factor during a machine-washing cycle. Indeed, according to Zaman et al. [9], a washing cycle may be decomposed into four damaging actions with possible interactions and interdependences:

- Mechanical stress (abrasion, flexion, hydrodynamic flow [16]);
- Thermal stress (temperature);
- Water stress;
- Chemical influence (detergent).

Indeed, as electronic parts are mostly made of metal, the chemical aspect should not be forgotten. Kellomäki et al. [10] reported the color change (from silvery to darker gray) of their silver-based antenna and explained it by the oxidation of silver. This phenomenon has not been much reported in the literature as the few studies on the washability of a silver material used bleach-free detergents [12] or even no detergent [17]. Only Slade et al. [13] were found to use a detergent with bleaching agents and concluded that metal-coated fabrics performed poorly after washing cycles. The specific responsibility of the bleaching agents, accounting for such results, was not highlighted. In this study, the mechanical and chemical impacts of the machine-washing process, on a silver-plated-nylon fabric used as a sensing skin electrode in a smart garment, are investigated separately and simultaneously. The impact of thermal stress has been investigated in another study presenting medical smart underwear, incorporating such silver-plated electrodes, which have to be washed in hospital laundries. To this purpose, fabric samples underwent different washing conditions with only mechanical action (machine-washing without detergent) or only chemical one (soaking in detergent) or both (machine-washed with different detergents). Then, the evolution of the visual aspect, the conductivity, the silver content and the surface composition of the washed fabric samples were analyzed and compared between the different washing conditions. In addition, silver-plated nylon yarns from different suppliers have been tested in each of those conditions to confirm that the results are valid for silver-plated yarns in general and are not specific to one supplier. New tools for characterizing metallic yarn such as spectroscopy, scanning electron microscopy and thermogravimetric analysis have been proposed and used. As a result, the mechanical and chemical impacts have been quantified separately but also simultaneously on the yarn properties such as lightness and electrical conductivity.

## 2. Materials and Methods

### 2.1. Realization of Textile Electrodes 

**Figure 1:**
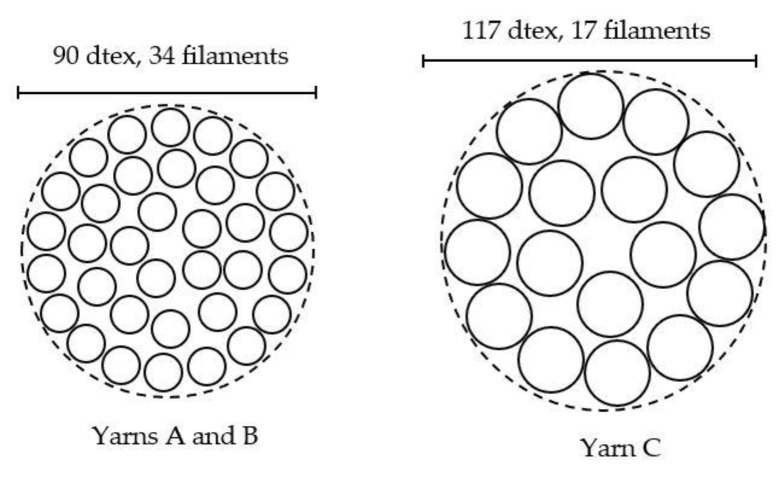
Yarns’ structures.

**Figure 2:**
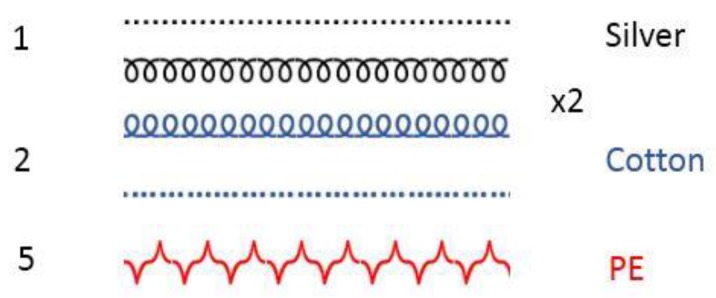
Diagrammatic representation of the fabric.

Silver-plated nylon yarns were purchased from three different suppliers. They will be referred to as Yarns A, B and C. Yarns A and B have a similar structure: 90dtex/34 filaments whereas Yarn C is thicker (117dtex) and made with only 17 filaments (Figure 1). Concerning the precise nature of the yarns, no details were given by the suppliers about the core nylon or the silver used for the plating. In addition, the metalizing process, which may impact the withstanding of the silver layer, was kept secret. Double jersey fabrics (back side in cotton) were produced with each yarn on an industrial circular knitting machine (CMO4A, Orizio). Figure 2 shows the diagrammatic representation of the knitting structure of the fabrics. Then, samples of 60 × 50 cm² and 5 × 36 cm² were cut off from each fabric in order to be washed. Those samples were then redivided during the washing process into two lots of smaller electrodes (30 electrodes of 10 × 10 cm² and 12 electrodes of 5 × 3 cm²) hence the chosen surface.

### 2.2. Washing Conditions 

**Figure 3:**
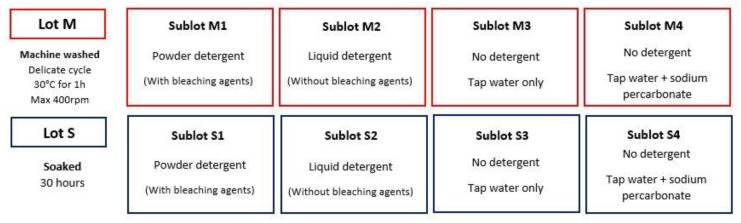
Schematic representation of the different washing conditions used for the study.

Figure 3 presents a schematic representation of different washing conditions. 

This study aims at evaluating the mechanical and chemical impacts of the washing separately and simultaneously, therefore each lot of electrodes was washed with two distinct processes: machine-washing, which was called Lot M, and soaking into a washing solution, which represents Lot S. Furthermore, those two lots were each subdivided into four sublots different by the detergent used. They are referred to by a number: 1 stands for powder detergent, which does contain bleaching agents (Omo Concentrated Professional, Diversey S.A.), 2 for liquid detergent (Eco Bulle Bio, Bulle Verte), which does not contain bleaching agent, 3 when no detergent was used in addition to tap water, 4 when sodium percarbonate (the fabulous sodium percarbonate, Starwax) was added and dissolved in tap water. As a result, the silver-plated-nylon electrodes from each yarn were split into eight sublots and washed separately in those eight different conditions. Figure 3 gives a visual synthesis of the experimental protocol. As this study was done for a garment manufacturer, the chosen washing conditions in the machine were those of its customers at their homes. For each sublots of Lot M, also referred to as Sublots M-X, the three large fabric samples (60 × 50 cm²) made of Yarns A, B and C were washed in a domestic washing machine (ENF 1486 EHW, Electrolux) with a delicate cycle (30 °C washing for an hour with wringing at 400 rpm) until 30 cycles with the corresponding detergents (Figure 3). For every washing cycle, 1.8 kg of random everyday clothes were put inside the machine with the samples to simulate real household washing, in compliance with the AATCC135 standard for laundry. For all Sublots M-X, square samples of 10 × 10 cm^2^ were cut off the 60 × 50 cm² samples of each fabric and withdrawn after every cycle until 30 cycles, then line dried. However, only the samples washed 5, 10, 15, 20, 25 and 30 times were deeply analyzed. Indeed, it turned out that measuring every sample of all sublots was time-consuming and not that meaningful as most of the measurements were very close, especially for Sublots M2 and M3. As a result, 18 samples (six per yarn) were analyzed for Sublot M-X. As recommended, 70 g of powder detergent and 60 mL of liquid detergent were used to wash samples from Lots M1 and M2, respectively. Lot M3 was washed in tap water without any detergent. For Lot M4, 7 g of sodium percarbonate was used. Powder detergents contain around 5% of bleaching agents. As the samples were washed with 70 g of powder detergent for Lot M1 (3.5 g bleaching agents), two times higher quantity of sodium percarbonate was used for M4. To evaluate the chemical action separately, the 5 × 36 cm² samples from the fabric A, B and C were cut in 12 smaller samples of 3 × 5 cm² and soaked in similar washing solutions as those of Sublots M-X inside the machine. However, as the volume of the soaking solution is different from the machine one, proportions of each detergent were recalculated. The samples of each fabric were soaked in 0.5 liters of tap water. The proportions preconized by the detergents’ manufacturers for hand washing, which are 35 g/5 L for powder detergent and 30 ml/5 L for liquid detergent, served as guidelines. Thus, 3.5 g of powder detergent, 3 mL of liquid detergent and 0 g of detergent were used to prepare the washing solutions of S1, S2 and S3, respectively. As 10% of the powder detergent mass of M1 was placed in M4, 10% of the 3.5 g put in S1, so 0.35 g of sodium percarbonate, were dissolved in tap water for S4. Since one washing cycle lasts one hour for Sublots M-X, the same soaking duration was taken for Sublots S-X. Therefore, samples of each fabric were withdrawn every 5 hours of soaking until 30 hours (corresponding to 30 machine cycles), then line dried. It turned out that studying only samples soaked 10, 20 and 30 hours was sufficient to study the chemical impact, as it is weak. As a result, 9 (3 × 3) samples were obtained for each sub lot. 

In a nutshell, 72 (18 × 4) electrodes were obtained from Lot M and 36 (4 × 9) from Lot S. All in all, 108 samples were analyzed, some deeper than others with the following tools. Thanks to these eight sublots, the mechanical and chemical impacts of the washing process can be highlighted and stigmatized separately but also simultaneously. Indeed, the sublots of Lot M were machine-washed whereas the sublots of Lot S were just soaked into a washing solution. Hence, mechanical actions of the washing machine are highlighted by comparing the results obtained by the respective sublots (M1 compared to S1 and so on). More specifically, the results of M3 show the isolated impact of mechanical stress as no detergents were used. Sublots of Lot S show the isolated impact of the chemical used with a specific focus on the bleaching agents, as they were presumed to have a damaging impact on the silver yarn. That is why their specific impact should be highlighted in washing condition 4 where only bleaching agents (sodium percarbonate), in higher quantity than contained in powder detergent, were dissolved in tap water. Sublot S3 aims at studying the withstanding water stress [9].

### 2.3. Color Evaluation 

As silver oxides and sulfides tend to be darker than pure silver, the lightness of the samples through washings was measured to reveal the potential presence of those oxides. Assessing the color change of textiles has already been used to determine the remaining coating material on the textile [18]. In this study, this method was used to estimate the potential surface oxidation of the conductive layer of the fabric. Color change of the silver-plated-nylon electrodes throughout the washing cycles was measured by using a spectrophotometer (CM-3610A, KONIC MINOLTA). The measurement conditions were set up with an illuminant D65 and 10° standard observer. The CIELAB (International Commission on Illumination) color space coordinates (L*: measurement of electrode lightness, a*: measurement of greenness to redness, b*: measurement for blueness to yellowness) were measured. Nevertheless, only the L* value (lightness) was taken into account for the discussion as it can be directly related to the surface oxidation as silver oxides are darker. All samples were measured five times to obtain a low standard deviation (SD < 1).

### 2.4. Electrical Characterization (Sheet Resistance)

Sheet resistance (also known as surface resistance or surface resistivity) characterizes the resistivity of a material divided by its thickness and represents the lateral resistance through a thin square of conducting material. Since it does not depend on the size of the tested material, a comparison between samples can be done easily. Thus, mechanical or chemical impact, of the washing process, on the electrical property of the silver-plated samples can be easily underlined by measuring this parameter.

**Figure 4:**
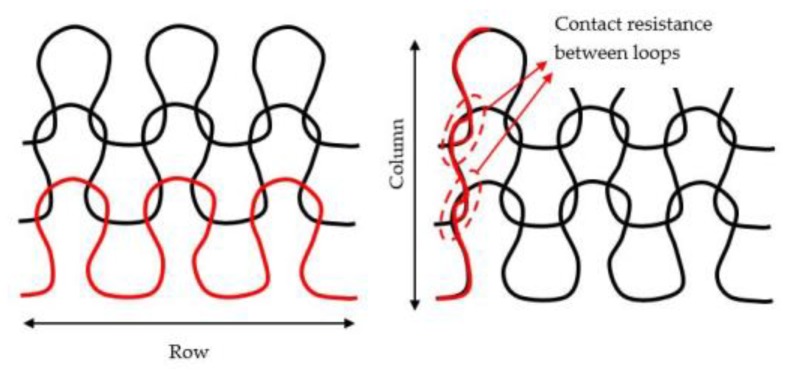
Current path through rows and columns.

A four-point probe system from Ossila was used for measuring the sheet resistance of the samples. This measurement uses four probes arrayed in a line, with equal spacing between each probe. A current is passed between the outer two probes, causing a reduction in voltage between the inner two probes, from which the sheet resistance can be calculated. As the samples are knitted fabric, the electric conductivity is not isotropic. Indeed, on rows, the current flow through a yarn but on columns it flows from loop to loop, creating a small contact resistance (Figure 4) as the contact between the filaments in the loop is not perfect. This resistance was quantified inferior to 1 Ω/loop by measuring linear resistance on different columns of the different samples. That is why, on each sample, measurements were done on rows and columns, then the mean was taken. For each sample, the sheet resistance was taken on three random locations in each row and three in columns to lower the standard deviation.

### 2.5. X-ray Photoelectron Spectroscopy (XPS)

To characterize the supposed presence of silver oxides on the silver layer’s surface, explaining the darker color, X-Ray Photoelectron Spectroscopy (Axis Ultra DLD, Kratos) was performed. X-Ray Photoelectron Spectroscopy is a surface analysis technique used to determine surface elements and their chemical states by analyzing the binding energy of photoelectrons, emitted from the sample surface illuminated with an X-ray source. Regarding the cost of such measurements, only 4 samples were analyzed: the reference sample of Yarn A, the 30-times-washed samples of M1, M2 and M4.

### 2.6. Scanning Electron Microscope (SEM) Observation 

In order to observe the damages of the silver layer on the conductive yarn, a scanning electron microscope (Phenom ProX, Thermo Fischer Scientific) was used. The identification of different chemical elements in the samples was accomplished with the Element Identification (EID) software package and a specially designed and fully integrated Energy Dispersive Spectrometer (EDS). The identification is aimed at determining the percentage of silver still present on the washed samples and the potential silver oxide that could have been formed during washing cycles. Reference sample (without washing) and samples washed, respectively, 10, 20 and 30 times for Lot M were observed and compared. Only samples soaked 30 hours were observed for Lot S.

### 2.7. Thermogravimetric Analysis (TGA)

To quantify the impact of the laundering on the silver content of the different fabrics, thermogravimetric analyses were carried out by a thermogravimeter (TGA 4000, Perkin Elmer). To improve the readability of the TGA curves and to obtain more accurate silver contents, silver-plated nylon yarns were unknitted from the fabric, which also contains cotton and polyester yarns (Figure 2). For each measurement, approximately 20 mg of silver yarn was put in the crucible and then heated from 30 °C to 900 °C in an oxygen atmosphere, then cooled down to 30 °C. This process took approximately 40 minutes per measurement. 

## 3. Results and Discussions

**Figure 5:**
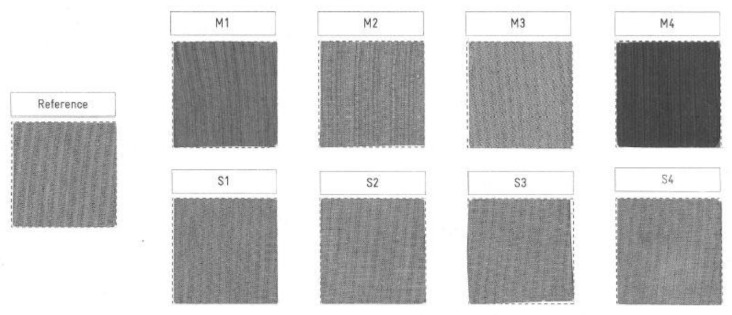
Aspects of 30-times-washed-electrode of each lot for Yarn A.

Regarding the visual aspect of the 30-times-washed electrodes (Yarn A) from the different lots, shown in Figure 5, the electrode from M4 has significantly darkened compared to the reference one and the others. All the electrodes from Lot S look similar to the reference one, as well as the M3 electrode.

**Figure 6:**
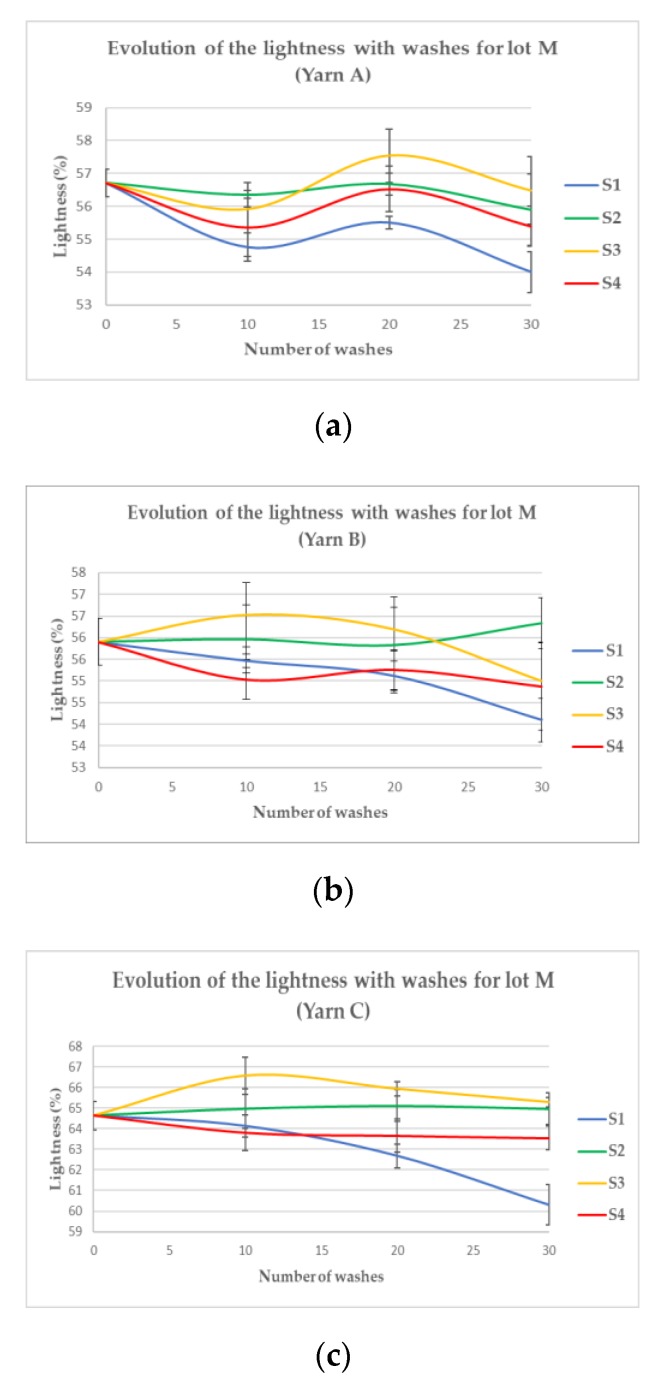
Evolution of the lightness with washes from Lot S, Yarns A (**a**), B (**b**) and C (**c**).

**Figure 7:**
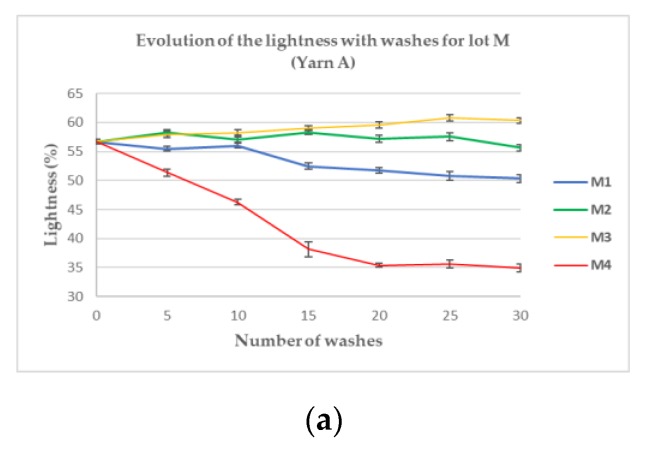

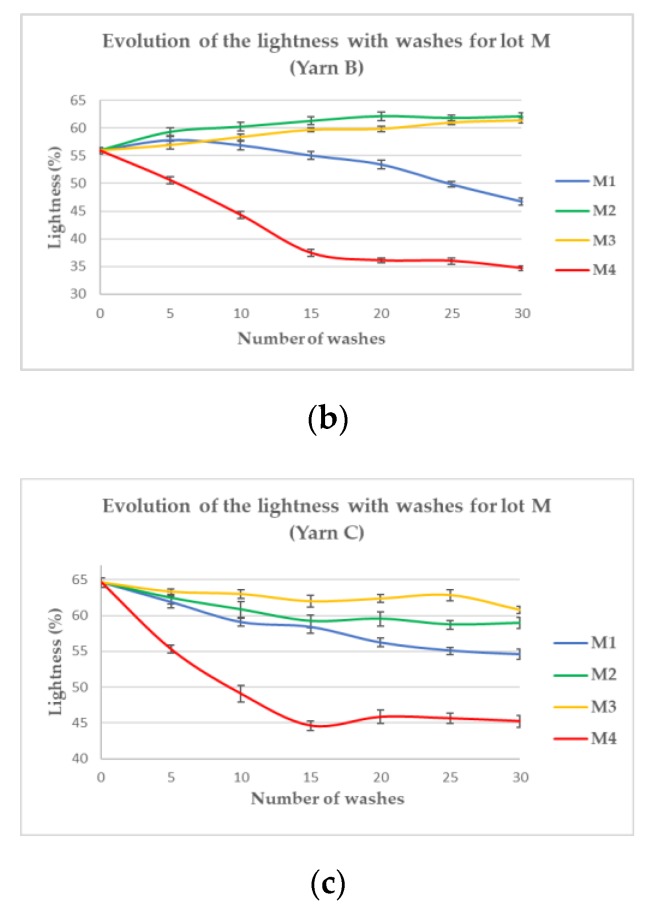
Evolution of the lightness with washes from Lot M, Yarns A (**a**), B (**b**) and C (**c**).

However, M1 appears also clearly darker and M2 slightly darker than the reference. Those visual differences were further quantified by a spectrophotometer throughout the 30 washing cycles. Resulting graphs for the three yarns studied are given Figure 6 and Figure 7.

Confirming the preliminary visual evaluation, the samples from Sublot M4 (Figure 6a–c) tend to darken rapidly and linearly until 15 (Yarn C) or 20 (Yarns A and B) washes. After this number of washes, the lightness remains constant, as if the phenomenon responsible for the darkening has reached its maximum. Samples from M1 tend also to darken gradually up to 30 washes but less significantly than M4 where the decrease is sharp. For Sublots M2 and M3, the variation of lightness does not show the same trend for every yarn; whereas it decreases for Yarn C in both washing conditions, samples made with Yarn B got brighter (+11.1% and +9.8%, respectively). Samples made with Yarn A became slightly darker (−3.2%) for M2 but brighter for M3 (+5.6%). To have a global vision of those trends for each sublot, the lightness variation between the samples washed 30 times and the reference one was calculated for each yarn and synthesized in Table 1.

Regarding the figures, all the yarns of M4 experienced a dramatic darkening, which is linear with a steep ratio until it reaches a minimum level (Figure 6). This level is very close for Yarns A and B (after a loss of approximately 38%, Table 1) and reached after 20 washes. It is interesting to note that their reference lightness was also very close (respectively, 57.1% and 55.9%). The reference lightness of Yarn C is higher (64.6%) than the two others and its minimum level is even more rapidly reached (after 15 washes compared to 20 for the others). Compared to the plummeting of lightness when washed with a high quantity of bleaching agents, the smaller quantity contained in powder detergents (M1) makes the lightness sink more slowly and steadily. Although the impact on lightness is not as noticeable as M4, it is nonetheless significant (around 14% on average). These results seem to indicate that bleaching agents tend to darken the yarns according to their concentrations. Indeed, samples from M2 and M3 of Yarns B and C, where no bleaching agents were used, do not unanimously show a darkening trend. Samples made with Yarn B have even got brighter in those washing conditions. Nonetheless, samples made with Yarn C have darkened no matter the washing conditions but half less for M1 than M4 and almost four times less for lot M2 and M3. Regarding the sublots washed in the machine (M1, M2, M3 and M4), the darkening seems to be directly related to the presence of bleaching agents in the water with a magnitude of the lightness decrease proportional to the quantity of bleaching agents introduced in the washing solution. Indeed, the more bleaching agents (M4), the darker the samples after 30 washes. A chemical reaction like oxidation of the silver layer can account for such a finding. All the more than silver oxides tend to be darker and less conductive than pure silver. Indeed, pure silver has an electrical conductivity of 6.30 × 10^7^ S/m whereas silver oxide (Ag_2_O) is a p-type semiconductor [18], whose conductivity is inferior by 10^3^ S/m. That is why an electrical characterization was also done on the machine-washed samples to determine if there was, in addition to the observed darkening, a loss of conductivity confirming the potential oxidation (see Table 2).

**Figure 8:**
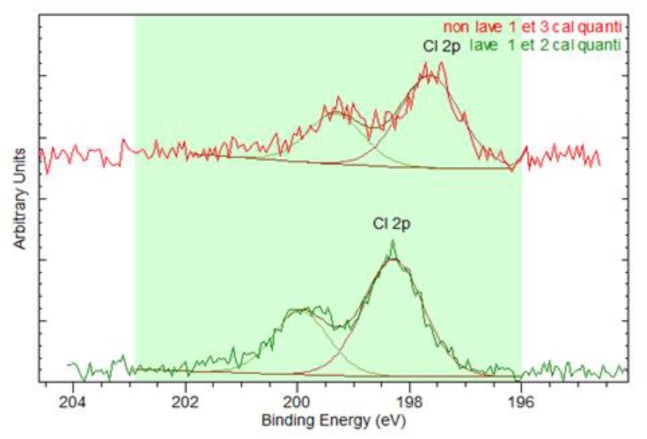
XPS spectrum of the chlorine detected on the electrode M1 yarn A.

Table 2 presents the evolution of the sheet resistance throughout the 30 washing cycles. A ratio between R_X_, representing the sheet resistance after x washes, and R_0_, which is the resistance of the reference sample, was calculated. Such a ratio appeared to make more sense than giving raw values as it shows directly the magnitude of the resistance increase. Results presented in Table 2 confirm that in addition to the previously observed lightness decrease, there is also a significant loss in conductivity. This is clearly evidenced by the lot M4, where samples’ resistance cannot be measured as it exceeds 10 MΩ, which is out of range of the multimeter used after 20 washes, no matter the yarn. Furthermore, for Sublot M1, for which there was also a slighter darkening, only the sample made of Yarn A presents a measurable resistance after 30 washes, but its resistance is extremely high regarding its reference one. For Sublots M2, the resistances of all the samples are quantifiable after 30 washes but their sheet resistance have also increased, even significantly for Yarn C. Globally, the resistance of this yarn is more impacted by the washing process because, in addition to a higher increase than the two others for Sublots M1, M2 and M4, a loss of conductivity is also observed when washed only with tap water (M3). Table 1 and Table 2 show a direct correlation between the darkening and the loss of conductivity. Except for Yarn B, which was washed with liquid detergent, the lightness has increased but the sheet resistance also, for which no explanation was found. The correlation seems to be so strong that the conductivity could be deducted directly by the lightness evolution without using a four-probe instrument. Definitely, with a darkening higher than 10%, the resistance of the electrodes is much probably higher than 10 MΩ. These findings seem to indicate the oxidation of the silver layer surface, turning into silver oxides. However, X-ray spectroscopy analysis, presented in Figure 8 and Table 3 and Table 4, shows that it is only partially the case. Indeed, the chlorine detected at the surface of the observed 30-times-washed electrode (Yarn A, M1) seems to be a metal chloride (Figure 8), regarding its binding energy around 198.5 eV [19]. It indicates that some silver chloride (AgCl) was formed. Indeed, according to Table 3, there is four times more chlorine after 30 washes with a powder detergent than on the reference sample (which has in fact been washed once after the knitting process, hence the presence of chlorine). This formation of silver chloride could have accounted for the previous findings. Looking closer at the values in Table 3, these silver oxides represent only 2.2%, which is meaningless compared to the decrease of the pure silver itself. Indeed, at the surface of the electrode, only half of the silver quantity still remains. This wiping out of silver is even more highlighted by Table 4 which presents the surface composition of the 30-times-washed-electrode from M4. Only 1.1% of silver remains at the surface of the analyzed zone of the electrode M4, Yarn A, which represents 7% of the silver quantity on the reference.

This significant depletion of silver, which is the conductive material, accounts for the previous results. Furthermore, if the observed darkening and loss of conductivity were only due to a chemical reaction like oxidation of the silver layer surface, electrodes from Lot S would also have darkened and lost conductivity dramatically. Results presented in Table 1 and Table 5 show that it is not the case.

**Figure 9:**
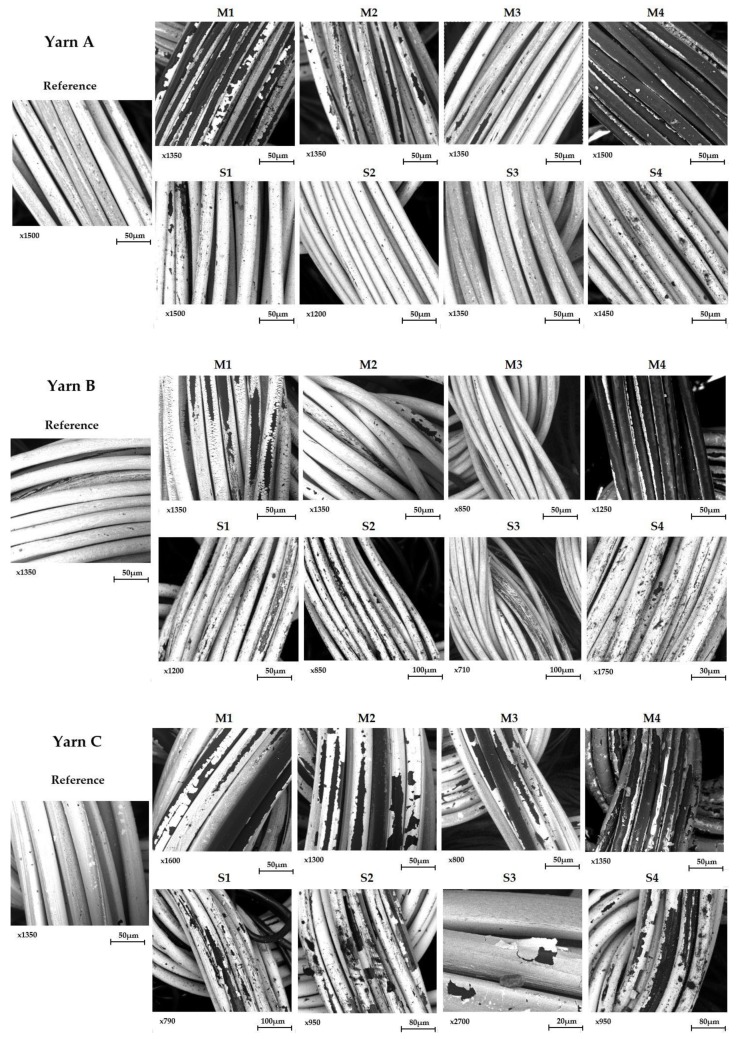
SEM observations of the electrodes made with Yarn A (**top**), Yarn B (**middle**) and Yarn C (**bottom**) for all the sublots.

Although a decrease in lightness (Figure 6 and Figure 7 and Table 1) and conductivity (Table 5) can be observed for Sublots S1 and S4 like for M1 and M4, the proportions have nothing in common. Indeed, the decline is barely significant apart from Yarn C, whose lightness dropped by 6.7% in S1 and sheet resistance was multiplied by almost four in S4. These results of the S-X sublots confirm the XPS analysis that the observed decrease in lightness and conductivity are not related only to the oxidation of the silver, turning into silver chloride but more probably to the possible removal of the silver layer from the core nylon yarn. This removal is clearly evidenced by the scanning electron microscope observations shown in Figure 9. 

These images explain well all the previous findings. Indeed, the loss of lightness and conductivity can be clearly related to the depletion, even the wipeout of the silver layer. Focusing on M4, there are no longer silver on the outside of Yarns A and B, hence they cannot be conductive anymore. Yarn C did not lose all its silver, explaining why its lightness decreases slighter than Yarns A and B, but still way too much to have a resistance lower than 10 MΩ. To account for the lightness that seemed to tend towards a lower limit after 20 washes, it was found on SEM images that all the exposed silver has already been removed after 20 washes. The measured lightness after 20 washes is one of the core nylons. The damaging of the silver layer also accounts for the results of M1, where the darkening and loss in conductivity were smaller. Indeed, the silver layer is seriously damaged. Some silver pieces still remain but they are isolated from one to another so the electrons cannot move. Such discontinuities in the silver layer are typically the scourge of Yarn B. For Sublot M1 where silver still remains, multiple small cracks in the conductive layer impeach the current to flow. Although a silver layer of samples from lot M2 presents some scratches, there is still continuity in the layer, making it able to keep a resistance lower than 10 MΩ even after 30 washes. Liquid detergents do also have an impact on the silver layer but in reasonable proportions. It is interesting to note that even washing 30 times with tap water in a washing machine damages the yarn (see M3 in Figure 9 for all the yarn). This is due to the rubbing against the drum of the washing machine and nothing can be done apart from reducing the spinning speed. That is why the delicate cycle has to be selected in order to reduce the number and intensity of the frictions against the drum. On the one hand, mechanical constraints of the washing machine do have an impact on the silver layer, to a further extent on the electrodes’ conductivity and performance. However, taken alone, this impact is very weak regarding the results of samples from Sublot M3. On the other hand, the chemical species contained in detergents alone have also a low impact as it can be seen on all the S-X images and results shown in previous tables. Even though, yarns from S4 and S1 present some damages their conductivity is still acceptable. However, when combined together, the simultaneous impact of chemical and mechanical actions can be devastating as seen on Sublot M4. Their cumulative effect is way superior to the sum of the impact taken separately. Eventually, even though oxidation of the silver layer is not directly observable at the surface of machine-washed electrodes, it does not mean that it did not occur. It means that oxidation has made oxidized elements more amorphous so more vulnerable to the mechanical constraints of the machine. It should be noted that contrary to Yarns A and B, which are barely not affected by tap water, the silver layer from Yarn C tends to delaminate, even in tap water, as shown in Figure 9. This phenomenon is too scarce to have a direct impact on the lightness and conductivity of the S3 electrode from Yarn C. Hence, it is not clearly noticeable in the S3 results presented by Table 1 and Table 5. Nonetheless, it can explain the overall poorer results of Yarn C compared to Yarns A and B in this study.

As these SEM images only analyze and show few mm² of an electrode, which is a hundred times larger, global conclusions cannot be drawn directly from these. That is why, to confirm what was visually observed qualitatively, a quantitative study was led. It consisted of quantifying the silver layer on each yarn by thermogravimetric analysis (TGA). As the degradation of nylon starts at around 330 °C and silver is not degraded till 900 °C, the weighed residue is only composed of pure silver at this temperature. It turned out that measurements have a high standard deviation. It can be explained because, on the one hand, the plating of silver is not strictly identical from one bobbin to another, and on the other hand, mechanical constraints from the washing drum are not equally applied on the fabric during the washing cycles. That is why a deeper analysis was done on Yarn A for investigating the impact of the bleaching agents on the silver content. Five successive measurements were done for each of the searched silver content (before washing and after being machine-washed 30 times). A comparison between yarns was also done, by measuring the silver content of each yarn for Sublots M-X. For Yarns B and C, only two measurements were done for each sample. 

**Figure 10:**
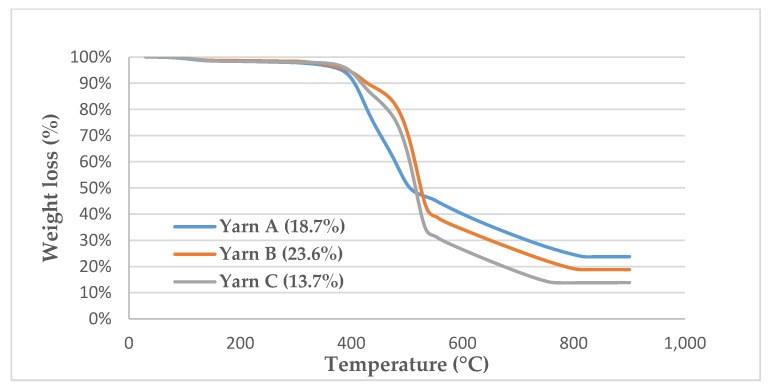
TGA curves of the reference Yarns A, B and C.

Firstly, the silver content of the reference electrode (before washing) from each yarn was measured in order to compare them (Figure 10). It turns out that Yarn B has a higher silver content and even contains almost twice more silver than Yarn C. It is interesting and reassuring to note that these three yarns were bought at three different prices, which in fact, respectively, varies according to their silver content. It would have also been tempting to link the silver content to the thickness of the silver layer, but it is not totally true. Indeed, a direct comparison cannot be drawn between Yarn C and the others as it is not perfectly similar in its construction. Whereas Yarns A and B have the same thickness and number of filaments, Yarn C is thicker but made with twice fewer filaments. As a result, the thickness of the silver layer cannot be compared directly with the measured silver content. It has neither been possible on SEM cross-section observations as the layer is so thin that the mechanical action of cutting the yarn distorted it. Nonetheless the withstanding to washes of the respective layers can be compared one to another by measuring the loss in silver content. A relatively high standard deviation was found for these measurements. Two phenomena can account for that. On the one hand, a small deviation is produced by the metalizing process itself. On the other hand, the fabric is made from different yarns coming from different bobbins, so they have overcome similar but not strictly identical mechanical constraints during the spooling, knitting and manual unknitting. The sum of those micro-constraints has a meaningful impact on the final silver content. Therefore, silver losses for Yarns A and B of Lot S cannot be distinguished from the measurement uncertainty that is why this quantification has only been done for Sublots M-X. Table 6 presents the different losses according to the washing conditions. As already highlighted by the previous results, the silver layer on Yarn C is globally less resistant than the other two yarns. The most striking difference is for the sublot washed only in tap water (M3) where Yarn C lost almost 18% of its silver content comparing to around 3% for Yarn A and less than 1% for Yarn B. The delamination of the silver layer occurs when the yarn is just soaked into water, shown in Figure 9. The image S3 for Yarn C from Figure 9 shows clearly such a result. It is also interesting to note that Yarn B is seriously stronger than Yarn A. As they have the same structure (thickness and number of filaments) their silver content reflects the thickness of their silver layer. According to the measurement of their respective reference sample, the silver layer of Yarn B is thicker than the one of Yarn A. Hence, the results of Table 6 indicate that the thicker the layer, the stronger. In other words, the thinner the layer, the more vulnerable to the constraints, which seems to intuitively make sense.

The silver depletion ratio was studied across the washing cycles to understand if the phenomenon is linear. In such cases, the critical number of washing after which the electrode is no longer conductive, hence no longer can functionality be predicted. As a result, only a few washes could be needed instead of 30 or more washes, which is time-and energy-consuming.

**Figure 11:**
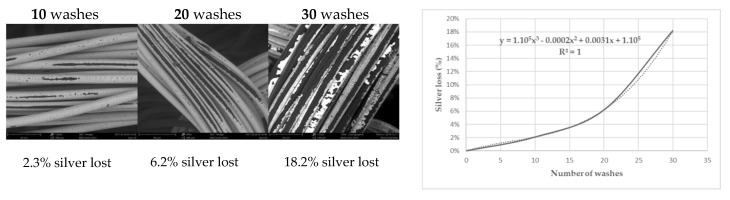
Evolution of the silver depletion with the washes, (**left**): SEM observations of the samples washed 10,20 and 30 times; (**right**): graph showing the silver loss according to the number of washes.

However, as evidenced by Figure 11, showing the silver depletion with the number of washes, the depletion is not linear but rather polynomial. It goes with the previous findings that the thinner the layer the more vulnerable. Indeed, each washing cycle tends to diminish the silver layer making this layer even more vulnerable for the washing cycles to come. 

## 4. Conclusions

By washing electrodes in various conditions, the mechanical and the chemical impacts of the washing process were studied separately and simultaneously. Indeed, one the one hand, samples of Sublot M3 have undergone only mechanical constraints of the washing machine as no detergent was added. On the other hand, samples for Sublots S-X were just soaked, so only the chemical impact was studied. Samples from M1, M2 and M4 were exposed to both effects but with different concentrations of bleaching agents, which were presumed to play a major role in damaging metal-plated-yarns. It turns out that taken separately neither mechanical constraints nor chemicals used have a significant impact on a silver-plated-nylon yarn on short terms (<30 washes). Indeed, even though SEM images show small scratches on the silver layer of M3 samples after 30 washes, its conductivity is still acceptable. Damages are even slighter on all the S-X sublots. However, the coupling of aggressive chemicals and the mechanical rubbings inside the machine can have a dramatic impact as seen on Sublots M1 and M4. More specifically, the impact of bleaching agents was highlighted. Their presence in the washing water, even the small quantity contained into powder detergents, damages seriously the silver layer, destroying the yarn’s conductivity so degrading the performance of the electrode within 30 washes. Regarding the high retail prices of smart garments, such a short lifetime is not acceptable for consumers. That is why liquid detergents, without bleaching agents, should be highly recommended by manufacturers for the laundering of smart garment integrating silver-plated-electrodes. Furthermore, in order to lower the mechanical rubbing of the drum, the delicate cycle with slower spinning speed should be advised. This study has also evidenced that there is a correlation between the prices of commercially available silver-plated nylons and their silver content, which impacts their withstanding to washing they withstand washing. Indeed, before washing, their electrical performances are quite equal as silver is very conductive even if the layer is thin. However, the thinner the layer, the more vulnerable it is towards washes. As a result, not surprisingly, the garment with a longer life would cost more, which is fine economically and interesting for consumers, and above all, more sustainable. Indeed, the release of silver in the wastewater is not suitable for aquatic organisms and their eco-systems, which is why it should be limited at best. Methods for quantifying the silver release in the laundry wastewater of smart textiles, integrating silver-plated nylon, should be further investigated. Furthermore, the chemical state of the released silver element should be clearly determined to evaluate its potential impact on the environment.

## Figures and Tables

**Table 1:** Lightness variation of the electrodes of different lots after 30 washes.

	M1	M2	M3	M4	S1	S2	S3	S4
Yarn A	−11.9%	−3.2%	5.6%	−39.0%	−5.5%	−2.2%	−1.1%	−3.1%
Yarn B	−16.4%	11.1%	9.8%	−38.0%	−3.2%	0.8%	−1.6%	−1.8%
Yarn C	−15.6%	−7.6%	−5.9%	−30.0%	−6.7%	0.5%	1.0%	−1.7%

**Table 2:** Evolution of the sheet resistance of the washed electrodes from different sublots (M1, M2, M3, M4).

Sublot	M1			M2			M3			M4		
Yarn	A	B	C	A	B	C	A	B	C	A	B	C
R_10_/R_0_	2.3	1.3	18.6	1.3	6.5	14.7	0.8	1.1	3.1	87.6	160.2	-
R_20_/R_0_	21.9	8.2	72,173	0.8	6.1	13.8	0.8	0.8	2.9	-	-	-
R_30_/R_0_	93,295	-	-	2.4	19.3	43.5	0.9	0.9	3.9	-	-	-

**Table 3:** Surface composition of M1 Yarn A.

	Ag	O	C	Cl	N
**Reference**	18.0%	15.9%	64.2%	0.5%	1.4%
**M1 Yarn A**	9.0%	13.1%	72.0%	2.2%	3.8%

**Table 4:** Surface composition of M4 Yarn A.

	Ag	Cl	O	C	Ca	N
**Reference**	15.7%	1.1%	13.2%	65.4%	0.0%	4.8%
**M4 Yarn A**	1.1%	1.1%	27.7%	57.3%	10.2%	2.9%

**Table 5:** Evolution of the sheet resistance of the washed electrodes from different sublots (S1, S2, S3, S4).

	S1			S2			S3			S4		
Yarn	A	B	C	A	B	C	A	B	C	A	B	C
R10/R0	1.30	1.43	1.25	0.87	0.84	1.03	0.96	0.84	0.90	1.25	1.51	2.01
R20/R0	1.33	1.88	1.24	0.92	1.05	0.92	0.82	0.83	0.76	1.50	1.47	2.81
R30/R0	1.47	1.61	1.47	0.88	0.90	0.83	0.91	1.10	0.89	1.13	1.37	3.86

**Table 6:** Comparison of the silver loss according to the washing conditions.

		Reference	M1	M2	M3	M4
Yarn A	Silver content	18.88% ± 0.26%	15.44% ± 0.34%	17.12% ± 0.27%	18.22% ± 0.34%	14.33% ± 0.15%
	Silver loss		**18.21%**	**9.32%**	**3.49%**	**24.12%**
Yarn B	Silver content	23.42% ± 0.11%	21.94% ± 0.14%	23.16% ± 0.12%	23.28% ± 0.05%	19.36% ± 0.10%
	Silver loss		**6.30%**	**1.10%**	**0.57%**	**17.31%**
Yarn C	Silver content	13.82% ± 0.14%	10.65% ± 0 17%	8.37% ± 0.21%	11.6% ± 0.02%	9.43% ± 0.37%
	Silver loss		**22.95%**	**39.43%**	**16.12%**	**31.77%**
